# Practicing Novel, Praxis-Like Movements: Physiological Effects of Repetition

**DOI:** 10.3389/fnhum.2016.00022

**Published:** 2016-02-05

**Authors:** Joshua B. Ewen, Ajay S. Pillai, Danielle McAuliffe, Balaji M. Lakshmanan, Katarina Ament, Mark Hallett, Nathan E. Crone, Stewart H. Mostofsky

**Affiliations:** ^1^Clinical Neurophysiology Laboratory, Department of Neurology and Developmental Medicine, Kennedy Krieger InstituteBaltimore, MD, USA; ^2^Department of Neurology, Johns Hopkins University School of MedicineBaltimore, MD, USA; ^3^Department of Psychological and Brain Sciences, Johns Hopkins University Krieger School of Arts and SciencesBaltimore, MD, USA; ^4^Center for Neurodevelopmental and Imaging Research, Kennedy Krieger InstituteBaltimore, MD, USA; ^5^Human Motor Control Section, National Institute of Neurological Disorders and Stroke, National Institutes of HealthBethesda, MD, USA; ^6^Department of Psychiatry and Behavioral Sciences, Johns Hopkins University School of MedicineBaltimore, MD, USA

**Keywords:** motor learning, attention, praxis, gesture production, EEG, event-related desynchronization, repetition suppression

## Abstract

Our primary goal was to develop and validate a task that could provide evidence about how humans learn praxis gestures, such as those involving the use of tools. To that end, we created a video-based task in which subjects view a model performing novel, meaningless one-handed actions with kinematics similar to praxis gestures. Subjects then imitated the movements with their right hand. Trials were repeated six times to examine practice effects. EEG was recorded during the task. As a control, subjects watched videos of a model performing a well-established (over learned) tool-use gesture. These gestures were also imitated six times. Demonstrating convergent validity, EEG measures of task-related cortical activation were similar in topography and frequency between the novel gesture task and the overlearned, praxis gesture task. As in studies assessing motor skill learning with simpler tasks, cortical activation during novel gesture learning decreased as the same gestures were repeated. In the control condition, repetition of overlearned tool-use gestures were also associated with reductions in activation, though to a lesser degree. Given that even overlearned, praxis gestures show constriction of EEG activity with repetition, it is possible that that attentional effects drive some of the repetition effects seen in EEG measures of activation during novel gesture repetition.

## Introduction

*Ideomotor praxis* refers to the performance of learned, skilled movements (Wheaton and Hallett, 2007). The loss of the ability to perform these actions—acquired apraxia—is well understood, but what is less well studied is how praxis skill develops in the first place. This question is of interest particularly in the study of developmental disabilities, including autism spectrum disorders (ASD), in which the ability to acquire praxis skill is often impaired (Mostofsky and Ewen, 2011). One potential avenue to study the ontogeny of praxis ability is to look at behavioral and physiological changes as an individual practices the performance of novel gestures. In particular, learning may occur by imitation, which is a critical target of research into the mechanisms by which infants learn a wide variety of skills (Paulus, 2014).

In this context, we use the term *gesture* to refer to a prespecified, skilled movement of the upper extremity. While some authors may use the term to refer only to movements that have meaning or function (e.g., goal-directed tool manipulation or communication), we use it here to indicate both meaningful and meaningless gestures, consistent with the usage of some other groups (Goldenberg et al., 2001; Pierpaoli et al., 2014; Vanbellingen et al., 2015).

The approach of studying praxis acquisition through novel gesture imitation, however, is not entirely straightforward, as there is some debate about the relationship between the neural systems underlying to the production of (overlearned, meaningful) praxis gestures and those underlying the production of novel, meaningless gestures. Liepmann, who is generally credited with the seminal description of the clinical syndrome of apraxia, regarded the overlearned nature of praxis gestures as central to the concept of praxis. Imitation of novel complex arm movements, which would be driven by external cues rather than internal representations, was therefore outside of Liepmann's conceptual view of praxis (Goldenberg, 2013). However, since that time, physiological studies have mostly demonstrated overlapping cortical activation during novel-gesture-imitation and praxis tasks (Makuuchi et al., 2005), though some authors suggest differential patterns of activation within the same networks during praxis vs. imitation tasks (Goldenberg, 2013; Hoeren et al., 2014).

At the core of the praxis network are the left posterior parietal and premotor (frontal) areas (Heilman and Valenstein, 2003; Wheaton and Hallett, 2007) as well as the white matter connections that span these two regions (Geschwind, 1965), however occipital, posterior temporal regions may also be involved (Goldenberg and Randerath, 2015). Primary visual cortex, V5, superior temporal sulcus, and primary motor cortex are also involved in praxis execution (Heilman and Valenstein, 2003), though they are not implicated via lesion studies as being specifically related to praxis. This left-hemisphere network is generally understood to be necessary and sufficient for praxis function, although homologous regions on the right are often activated (to a lesser degree) by praxis performance in physiological studies (Moll et al., 2000; Wheaton et al., 2009; Ewen et al., 2015) and right-sided lesions sometimes can lead to clinical symptoms (Wheaton and Hallett, 2007).

The experimental paradigm which we devised for this study, as described below, relies on within-session practice of novel, meaningless gestures that are kinematically similar to praxis gestures. Our first goal was therefore to demonstrate that similar networks are activated in the performance of praxis gestures and the imitation of novel gestures. Although EEG does not generally have the spatial resolution to identify specific cortical sources without source localization analysis, we anticipated seeing similar scalp patterns reflective of activation of the praxis network both during praxis execution and during the imitation of novel gestures.

Our second goal was to assess the effects of repetition on the imitation of novel gestures. Whereas, the (one-time) imitation of gestures has been studied, *learning* of gestures has been less well characterized. To develop a task that would examine the learning of gestures, we turn to the broader literature related to motor skill learning as it relates to other, non-praxis tasks. Results here appear to be highly dependent on the paradigm used, and most use movements that are fairly simple, kinematically (Kelly and Garavan, 2005). As a result of this task specificity, studying learning effects relevant to the praxis network requires a task that causes the production of praxis-like gestures; more commonly used learning tasks, such as pinch force, are unlikely to give results that one can confidently apply to the study of the praxis network.

What effects might we expect with practice? Much of the existing literature on motor learning reveals a reduction in the topographical area and magnitude of activation over the course of practice (Kelly and Garavan, 2005). In sensory tasks, effect of decreased physiological activation associated with repeated exposure is often referred to as “repetition suppression” (Krekelberg et al., 2006). This decrease in amount of neural tissue activated has been interpreted as resulting from an increase in cortical “efficiency” (Poldrack, 2000; Kelly and Garavan, 2005; however, see also Poldrack, 2015).

The vast majority of physiological studies examining motor practice have been performed with fMRI. Whereas, fMRI has better spatial resolution, EEG has an advantage in terms of measuring directly the electrical activity produced by ensembles of neurons that is involved in brain-based computation. Differential effects on varying frequency components allow EEG studies to dissect phenomena that may be conflated in the BOLD signal. Alpha (7–12 Hz) and beta (13–30 Hz) rhythms each have relevance both to motor function and attentional deployment. Alpha in central regions is typically referred to as the “mu rhythm.” Mu activity is observed when the motor system is at rest, and suppression (or event-related desynchronization; ERD) of mu is associated with cortical activation during movement as well as motor observation and imagery (Pfurtscheller and Neuper, 1994). As mu is one of the best described phenomena in EEG, mu ERD represented our primary indicator of interest for assessing practice effects in the novel gestures and control task. Alpha activity is also generated from visual areas, and posterior alpha ERD is associated with visual attention (Ikkai et al., 2014).

Beta rhythms are also well described in the motor system, with generators throughout cortical and sub-cortical levels (Baker et al., 1999; Wheaton et al., 2005; Neuper et al., 2006; Engel and Fries, 2010). As with alpha, beta ERD is typically associated with movement. Motor effects on beta activity are typically seen in the higher range of the beta band (~18–30 Hz). Posterior beta ERD within the visual system has also been associated with attentional manipulation (Palva and Palva, 2007). Magnitude of both alpha and beta ERD, then, can be used to assess movement- and attention-related aspects of motor skill performance (Nakano et al., 2013; Gonzalez-Rosa et al., 2015).

By examining modulations of alpha and beta ERD, we sought to validate this novel-gesture-learning task as a model of praxis-like gesture learning. There were three sub-goals: (1) to demonstrate convergent validity between a new meaningless gesture imitation task and an overlearned-praxis-gesture task, as measured by scalp-EEG-based indices of cortical activation; (2) to determine whether EEG measures of activation show repetition-suppression-like effects; and (3) to assess whether physiological changes associated with the practice of novel gestures are qualitatively or quantitatively different from the changes associated with repetition of previously learned praxis gestures. We hypothesized (1) that the novel gesture task would elicit EEG activity similar to that elicited by the praxis task, consistent with the notion that similar networks are being activated in the production of gestures in these two experimental contexts. We also hypothesized (2) that, over the course of repetition, the novel gesture imitation task would elicit decreasing amounts of alpha ERD, consistent with a notion of increasing “efficiency” in the task-relevant network. Finally, we hypothesized (3) that, while the praxis task would evoke progressively decreasing amounts of alpha ERD associated with decreased attentional demands, the change (ERD decrease) seen would be less than that seen for the novel gesture task.

## Method

### Subjects

Thirteen neuro-typical adults (six males), aged 18–45 years (mean = 25.5; SD = 7.67) participated in the study. All were right-handed by self-report. We excluded individuals with any neurologic or psychiatric diagnoses, or who were taking any neurologically active medication. Written informed consent was obtained on all subjects. Participants were compensated for their participation. This study was approved by the Johns Hopkins Medicine IRB and the Kennedy Krieger Institute Pre-IRB Review Board.

### Task—novel gestures (NG)

**Figure 1:**
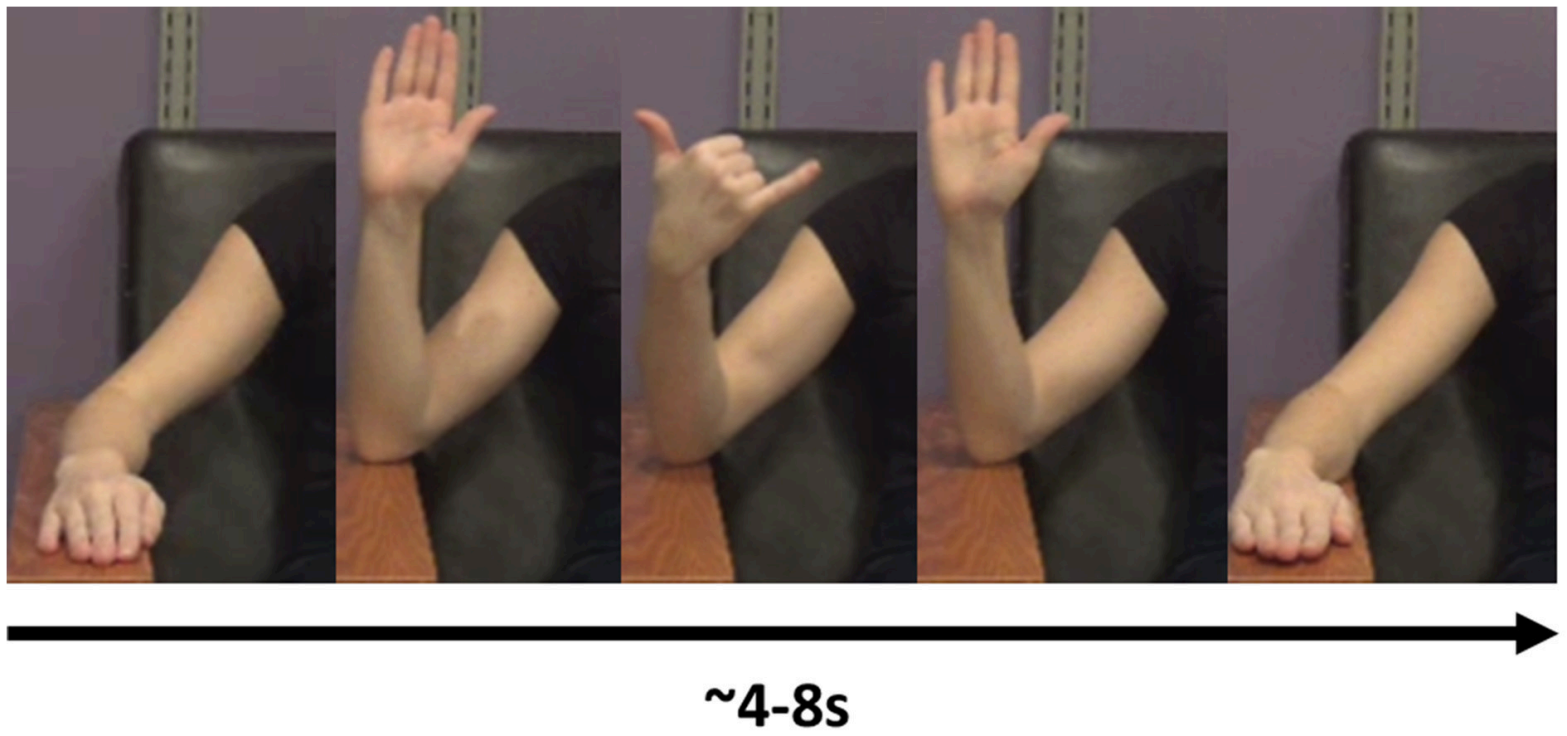
**An example of a novel gesure**. In this gesture, the model flexes the elbow, then flexes the middle three fingers while supinating at the wrist. She then extends the three middle fingers and extends at the elbow, back at the resting position.

The task consisted of the imitation of 16 different meaningless gestures. These gestures were designed to be kinematically similar to praxis gestures in the following sense: the gestures involved the fluid performance of a sequence of movements with the forearm and hand (see “NG Videos 1 and 2” in Supplemental Materials). Additionally, the gestures also involved simultaneous performance of movements with multiple joints. All movements were constrained by the requirement to keep the elbow on the armrest, so as to decrease the impact of movement artifact on the EEG signal. An example gesture included a sequence such as the following: Start with the hand in a neutral, pronated position, resting on the arm-rest. Flex at the elbow while approxmiating the thumb and second finger. Then, while extending the elbow, extend the second finger while flexing the third and fourth fingers to approximate the thumb (Figure 1; “NG Video 1” in Supplemental Materials).

**Figure 2:**
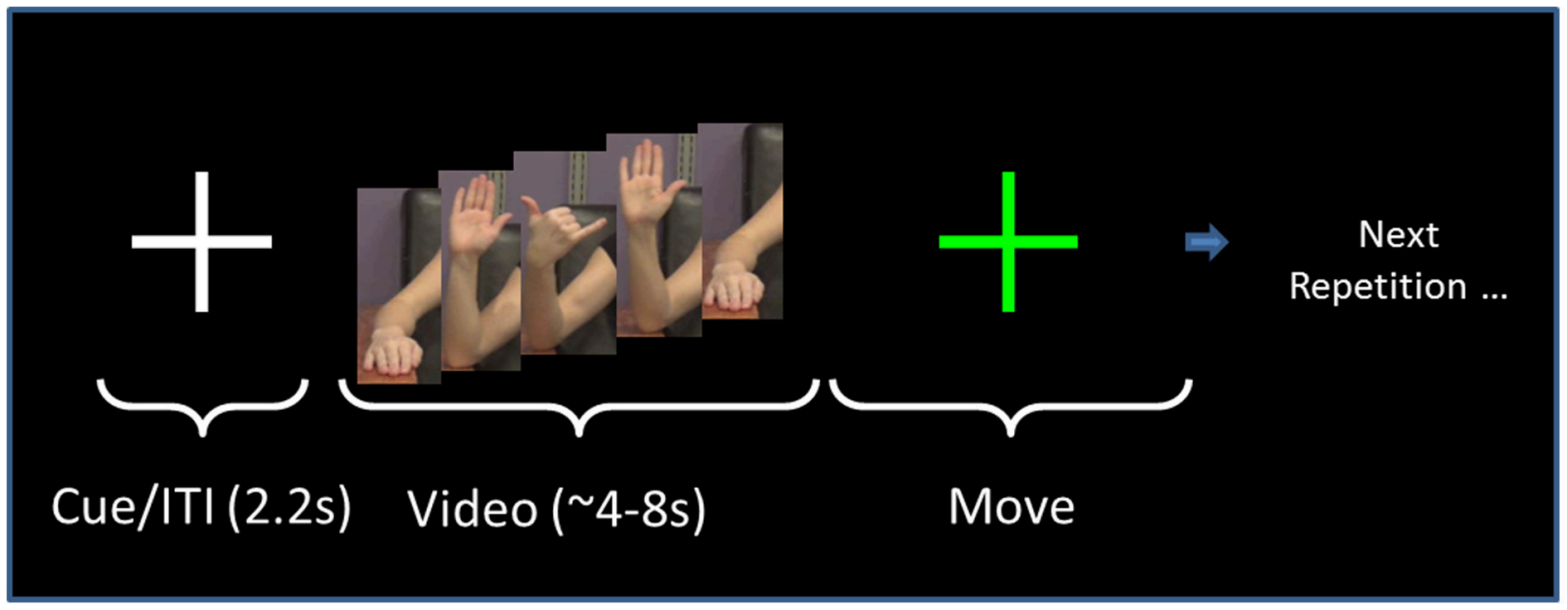
**Task sequence**. The order of presentation for both NG and PX tasks consists of a fixation cross that is displayed for 2.2 s. This is followed by the video, which lasts for 4–8 s, depending on the specific gesture. The subject performs the gesture for a duration approximately equal to the video length. The researcher then gives performance feedback. The same gesture (video/imitation) is repeated six times as above. The task then moves to the next gesture.

The format of the presentation was the same for all gestures (Figure 2). Following a fixation cross that lasted 2.2 s, the screen showed the right arm of a model performing a gesture (Figure 1), as described in the following paragraph. The model performed the gesture two times. We chose to have the model perform the gesture two times to have a relatively large block of time on which to perform the analysis. Videos of the model perfoming the gesture lasted from 4 to 8 s. Once the video concluded, the screen showed a “Go” cue. At this point, the subject imitated the performance of the gesture with his/her right arm (two times, as in the video). This performance was considered *Repetition 1*. A research assistant observed the subject performing the gesture and scored the gesture repetition as correct or incorrect. Following the conclusion of *Repetition 1*, the research assistant gave feedback to the subject as to whether he/she perfomed the gesture correctly. If the subject performed the gesture incorrectly, then the research assistant gave feedback such as, “Notice how the model moves her fingers.” This form of feedback was selected as being sufficiently vague so as to spur the subject to watch the video closely to improve performance. It was felt that an absence of feedback could result in subjects inadvertently committing the same error throughout the repetitions.

The subject next watched the same video again, and, on the “Go” cue, performed the gesture again. This was *Repetition 2*. The research assistant again provided feedback. The same gesture was repeated for a total of six repetitions. After the sixth repetition, the task moved to the next gesture. Hereafter, the visual (video-watching) portion of *Repetition 1* is referred to as “V1.” The motor execution portion of *Repetition 1* is referred to as “M1.”

### Task—praxis (PX)

As a control condition, we had subjects pantomime the use of 12 tools, including a hammer, screwdriver, doorknob, and pen (Videos 3 and 4 in Supplemental Materials). The overall format of video presentation and execution were the same as for the NG task. The model in the videos was shown holding the object in question, so as to emphasize the semantic aspects of the praxis movement. As in the NG tasks, the video and imitation were repeated six times.

### EEG recording

**Figure 3:**
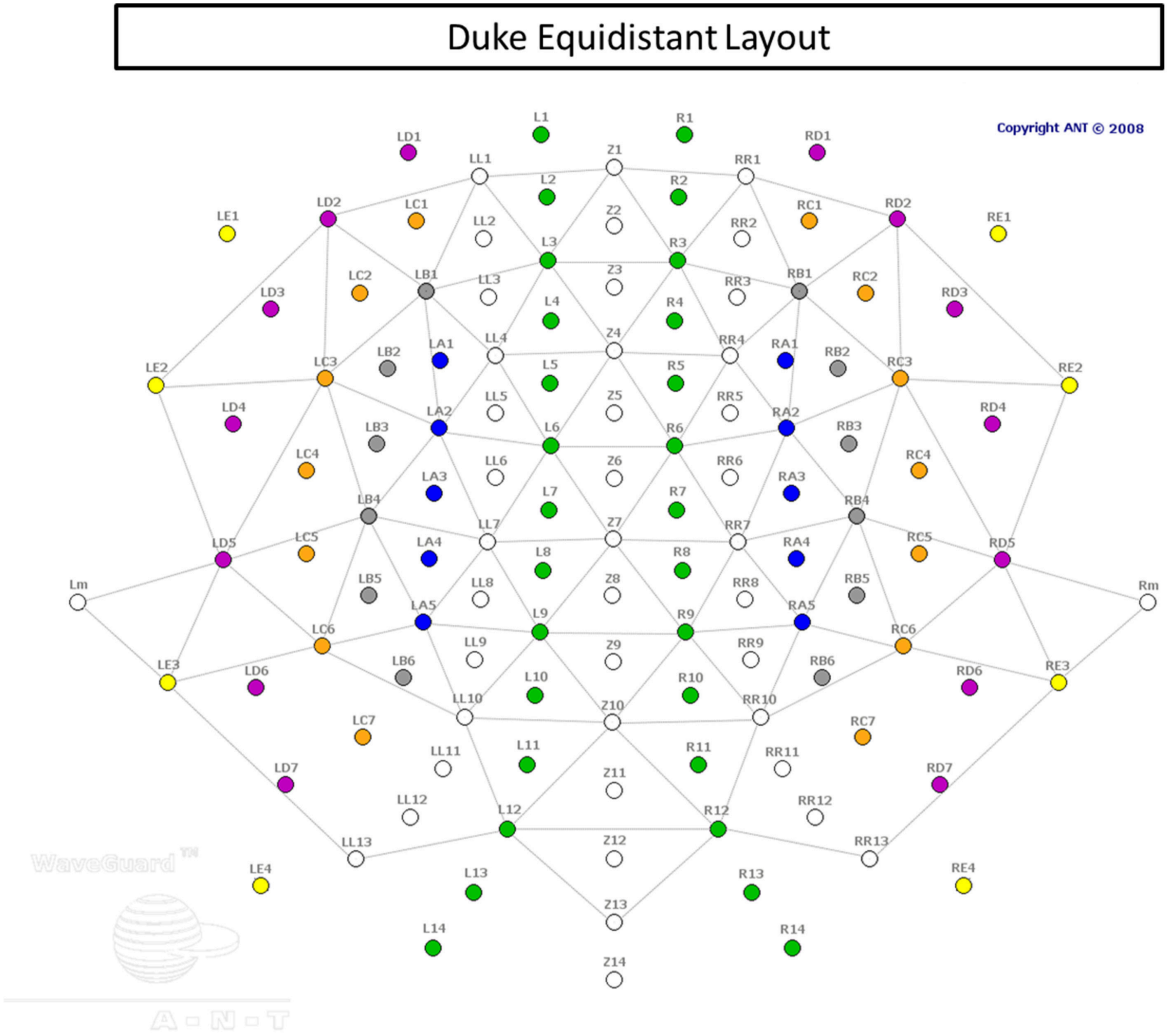
**Electrode cap layout**.

EEG signals were recorded from a 128-channel WaveGuard cap (Figure 3), with equidistant coverage of the entire scalp (Duke montage; Advanced Neuro Technologies [ANT], the Netherlands). The cap used an active shielding technology. The recording was conducted with an *ASA* amplifier (ANT, the Netherlands), from DC to 138 Hz (hardware anti-aliasing filter), at a sampling rate of 512 Hz, with an average reference as the recording reference. Electrode impedances were kept below 10 kΩ in all channels.

### EEG pre-processing

Signal pre-processing was performed using ANT *asa-lab* software. The data were filtered using a high pass filter with a cutoff of 0.2 Hz and slope of 24 dB/Oct. Artifact correction for eyeblinks, lateral eye movements, and stereotypical muscle activity, such as jaw clenching was performed using a PCA-based method within the *asa-lab* software. This artifact correction algorthm uses principal component analysis (PCA) to remove artifacts by removing those principal components which are most tightly associated with multiple instances of the artifact and leaving the remaining signal, which is believed to represent the artifact-free signal. We remove principal components which account for ≥95% of the variance associated with the selected several instances of the type of artifact.

Several eyeblinks, lateral eye movements, and jaw clenches were visually identified based upon well-defined morphology on a subject-by-subject basis. Eye blinks can be defined by brief, subject-dependent, monopolar potentials. Lateral eye movement can be identified by a longer durations of singnificantly increased amplitude potentials, but give bipolar, negative, and positive potentials in the RE1 and LE1 channels, based on direction of movement. Muscular and movement artifacts (such as jaw clenches) were identified by visually inspecting the signal and also by video verification. After correction we did not have to remove any trials.

All EEG signals were visually inspected, and channels with persistent artifact were removed. Only one channel was removed from analysis out of all subjects' data. If a channel was removed from a particular subject, his/her average reference was updated to reflect the absence of that channel.

To minimize spatial blurring due to volume conduction, signals were converted from average reference to current source density (CSD) estimates, computed using the spherical spline algorithm (Kayser and Tenke, 2006).

We next epoched the video segment from the onset of the video to the onset of the “Go” cue. We also epoched the movements based on review of the video.

### ERSP analysis

*Event-related spectral* perturbations (ERSP) refer to changes in the amplitude of a certain frequency component of the EEG signal related to a task. Task-related increases in a frequency are referred to as *event-related synchronization* (ERS), while task-related decreases are referred to as *event-related desynchronization* (ERD). In most cases, it is ERD and not ERS that is associated with task-related increases in cortical function. Both alpha (Pfurtscheller et al., 1996a; Kelly et al., 2006; Ikkai et al., 2014) and beta (Pfurtscheller et al., 1996b; Engel and Fries, 2010) have been viewed as either inhibitory or as a cortical “idling rhythm.” As such, alpha and beta ERD often accompany an increase in activity. Alpha ERD has been linked to an increase in metabolic activity (Oishi et al., 2007). One specific example of this effect include posterior (occipital) alpha, which exists in the visual cortex and is enhanced (ERS) during the closing of eyes and suppressed (ERD) when eyes are opened. Mu activity, on the other hand, refers to a central rhythm that eminates from primary sensori-motor cortex and includes both alpha and beta components (Hari, 2006). Mu suppression (ERD) occurrs during movement of the contralateral body as well as during movement observation and imagination (Pfurtscheller and Neuper, 1994; Neuper et al., 2006).

The EEG is a multidimensional signal, with results spanning topography (different electrodes/channels), frequency space, different parts of the experiment (baseline, visual, and movement × repetitions 1 + 6), individual subjects and different gestures. For the purposes of statistical testing, we developed four measures for different parts of the task (V1, M1, V6, and M6), for each subject. These analyses collapsed (averaged) across individual gestures. The workflow was as follows: (1) compute the power in active and baseline portions of the EEG signal, in all frequencies and all channels, averaging together all gestures; (2) in alpha and beta bands, compute ERSP in the active portions (visual and movement) as a *z*-score relative to the baseline's mean/variance; (3) from the 128 channels, interpolate topographical maps with a resolution of 250 × 250 pixels; (4) produce a grand average scalp map by averaging all subjects' *z*-score data; (5) calculate the maximal *z*-score value in the grand average and take 50% of this value; (6) then, in each individual subject, sum together all ERSP *z*-score values that are greater than the 50% value calculated from the grand average. This results in an integrated value for each subject. This pipeline was repeated individually for V1, M1, V6, and M6.

ERSP analysis was conducted in EEGLAB (Delorme and Makeig, 2004; MATLAB, The Mathworks, Natick, MA). Using wavelet decomposion, we assessed power in two active portions of the task: the visual portion, which included all time points from the onset to offset of the video; and the movement portion, which included all time points from the initiation of the movement (flexion of the elbow) to offset of the movement (return of the forearm back to the armrest). The onset and offset of motor activity were assessed via onset of signal from biceps brachii EMG.

The analysis to this point has measured the power in the signal of the task-active portion of the EEG signal. To assess ERSP, we next measured the power in the baseline, which consisted of the 1 s during the display of the fixation cross. In each frequency band of 1 Hz width, we computed the power of the signal for a time period that is adaptive to the frequency. For each frequency band, we then computed the ERSP of the active portion of the recording (movement and visual) as a *z*-score computed using the difference in means between the baseline and active portion amplitude measurements, divided by the variance of the baseline amplitude measurements. Examining spectrograms, we found the strongest ERD effects in alpha (7–12 Hz) and the higher, “motor” range of beta (18–25 Hz). Further analyses examined ERD in each of these two frequency ranges separately.

This procedure was performed in each channel, for measurements in V1, V6, M1, and M6, both in the novel getures (NG) and praxis (PX) tasks. We then interpolated values linearly for a scalp map of 250 × 250 pixels.

Our outcome measures of interest were the ERSP measurements as integrated topographically over active regions of the scalp. In order to define borders for our analysis, we performed the following procedure: using the grand average individually for alpha and beta, in each task phase (V1, V6, M1, M6) we found the topographical point of greatest ERSP *z*-score. We then drew continuous curves, individually for each subject and each task phase, around all points on the scalp map that had a value ≥50% of the grand average maximal ERSP *z*-score. We then integrated all ERSP values contained within all of the curves.

To test our prediction that the amount of activation (ERD) would decrease to a greater degree in the NG task as compared with the PX task, from V1 to V6 and also from M1 to M6, we performed four repeated measure, 2 × 2 ANOVAs: one each for alpha-video, beta-video, alpha-movement, and beta-movement, examining for the repetition (1 vs. 6) by block type (NG vs. PX) interaction effects. We then conducted *post*-*hoc* paired *t*-tests to verify the presence of task-related ERD decreases from repetition 1–6 in alpha-video, beta-video, alpha-movement, and beta-movement.

## Results

### Behavioral results

Averaging over both subjects and gestures, the participants performed the novel gestures correctly in an average of 2.16 ± 1.71 attempts (mean ± SD). There was some difference in the apparent difficulty of the different gestures: the easiest gesture was performed correctly in an average of 1.15 repetitions, across subjects, and the most difficult was performed correctly in an average of 5.31 repetitions. There was, however, relatively little difference across subjects: the best-perforning subject imitated the novel gestures correctly in an average of 1.2 repetitions, whereas the worst-performing subject imitated the gestures correctly in an average of 2.88 attempts. Errors included spatial errors, such as incorrect direction of wrist rotation and incorrect finger position, and sequence errors (e.g., with hand postures).

In the praxis task, all subjects performed all gestures correctly in all repetitions.

### ERD results

#### General ERSP results

**Figure 4:**
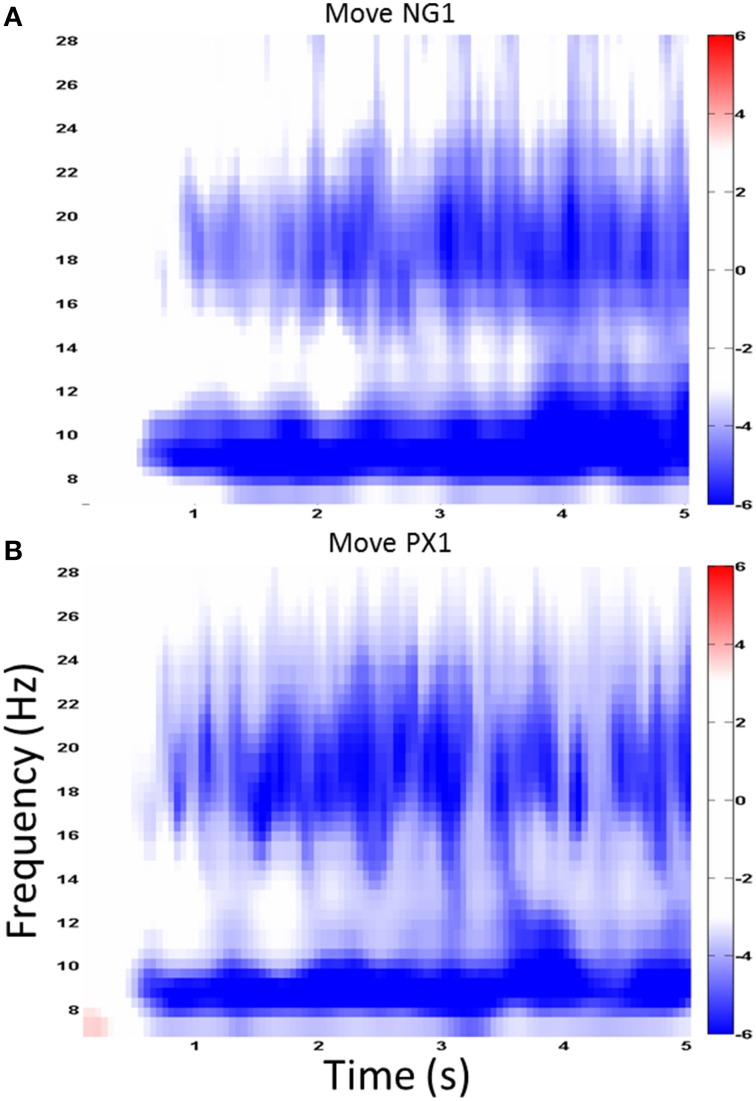
**Wide-band ERD spectrogram**. Similar ERD results from a central channel in the NG **(A)** and PX **(B)** tasks. The greatest ERD effect is seen in alpha (7–12 Hz) and “high” beta (18–25 Hz).

**Figure 5:**
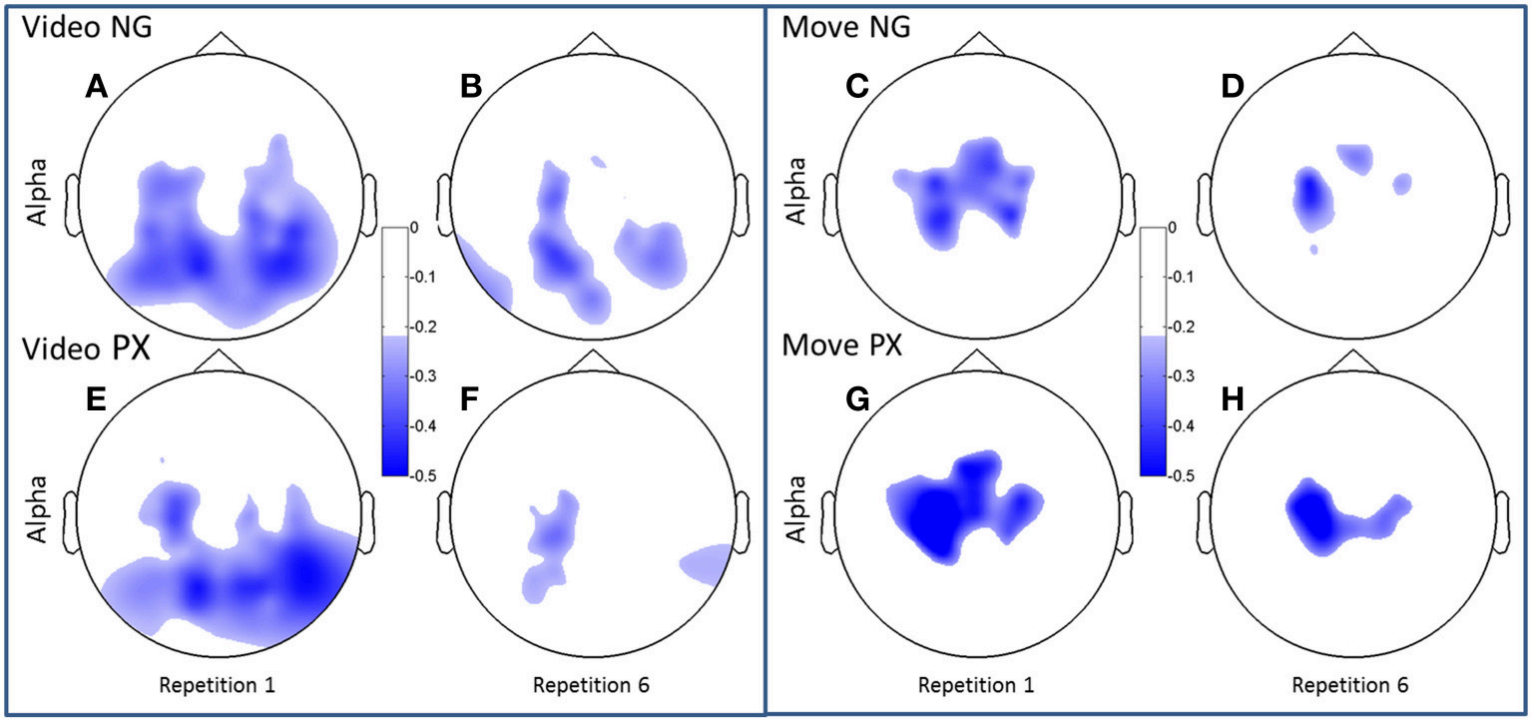
**Topographical plots of ERD (alpha band)**. Scalp plots are shown for NG **(A–D)** and PX **(E–H)** in the alpha band, for video **(A,B,E,F)** and movement **(C,D,G,H)**, for repetitions 1 **(A,C,E,G)** and 6 **(B,D,F,H)**. The overall topography of alpha ERD is similar between tasks (NG and PX). NG showed significant decreases in amount of ERD in both visual and movement task phases; PX showed significant changes only in movement.

**Figure 6:**
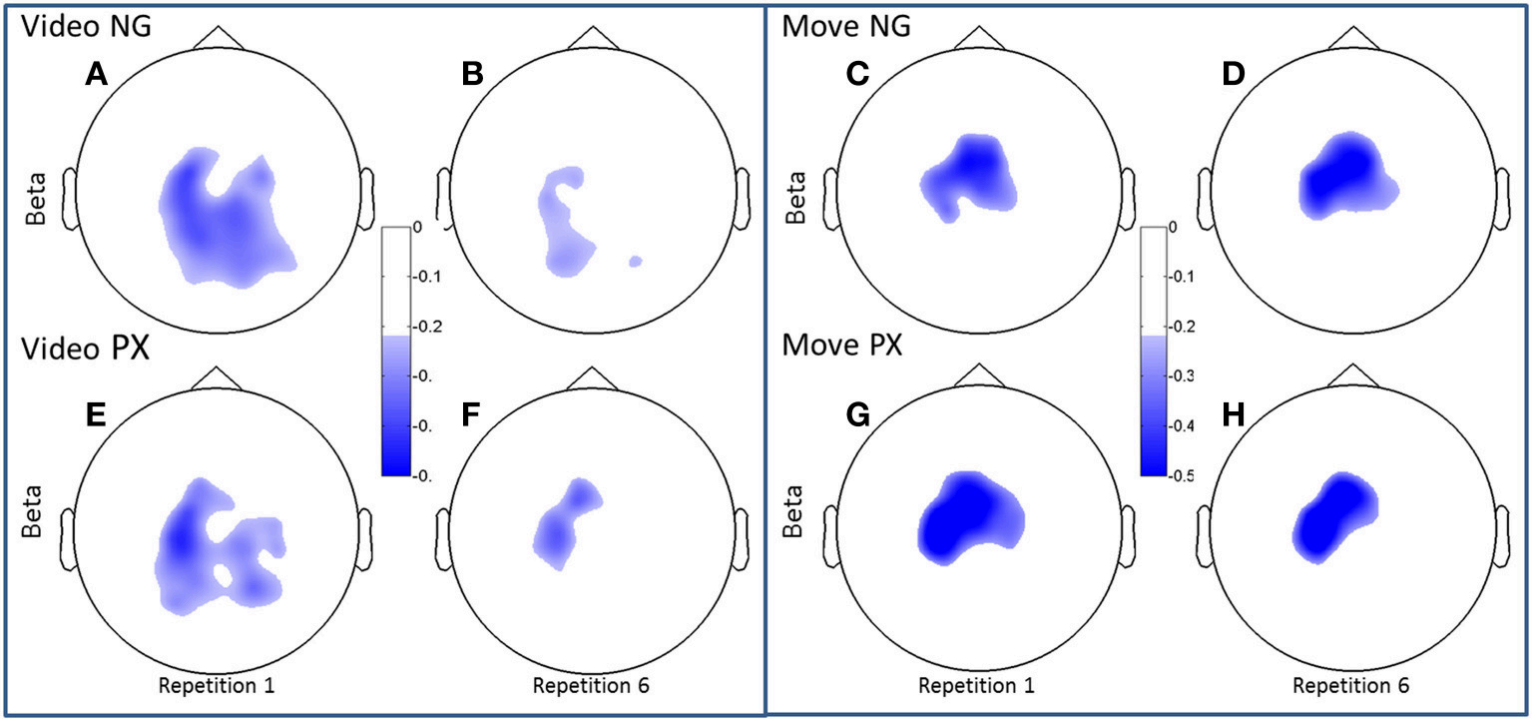
**Topographical plots of ERD (beta band)**. Scalp plots are shown for NG **(A–D)** and PX **(E–H)** in the beta band, for video **(A,B,E,F)** and movement **(C,D,G,H)**, for repetitions 1 **(A,C,E,G)** and 6 **(B,D,F,H)**. In beta, both NG and PX showed significant ERD decreases in movement, whereas there were no significant changes in movement in either task.

We first examined ERSP in the novel gesture data in a wide frequency band. The analysis showed ERD within the alpha band (7–12 Hz) and in a high beta band (18–25 Hz), which has been associated with motor processes (Wheaton et al., 2005; Neuper et al., 2006; Figure 4). We next examined the topographical spread of the alpha ERD. During observation of novel gestures (NG-V1), alpha ERD occurred in posterior scalp regions as well as bilateral central scalp regions, while beta ERD occurred a similar distribution, thought slightly more restricted than alpha (Figure 5). During the motor execution of novel gestures (NG-M1), alpha ERD was seen in left, right, and midline central regions. Beta again had a similar distribution but was somewhat more restricted topographically (Figure 6). The regions of activation were overall similar to those seen in prior EEG studies of praxis performance (Ewen et al., 2015).

#### Comparison of ERD in novel gesture vs. praxis tasks

Both alpha and beta ERD in the control praxis task were seen in similar regions to the corresponding analyses in the novel gestures task (Figures 5, 6). These results substantiate the contention that the novel gesture task activates brain mechanisms with similar physiology, in topography and frequency space, as the overlearned praxis gesture task.

#### Changes in ERD over the course of practice/repetition

**Figure 7:**
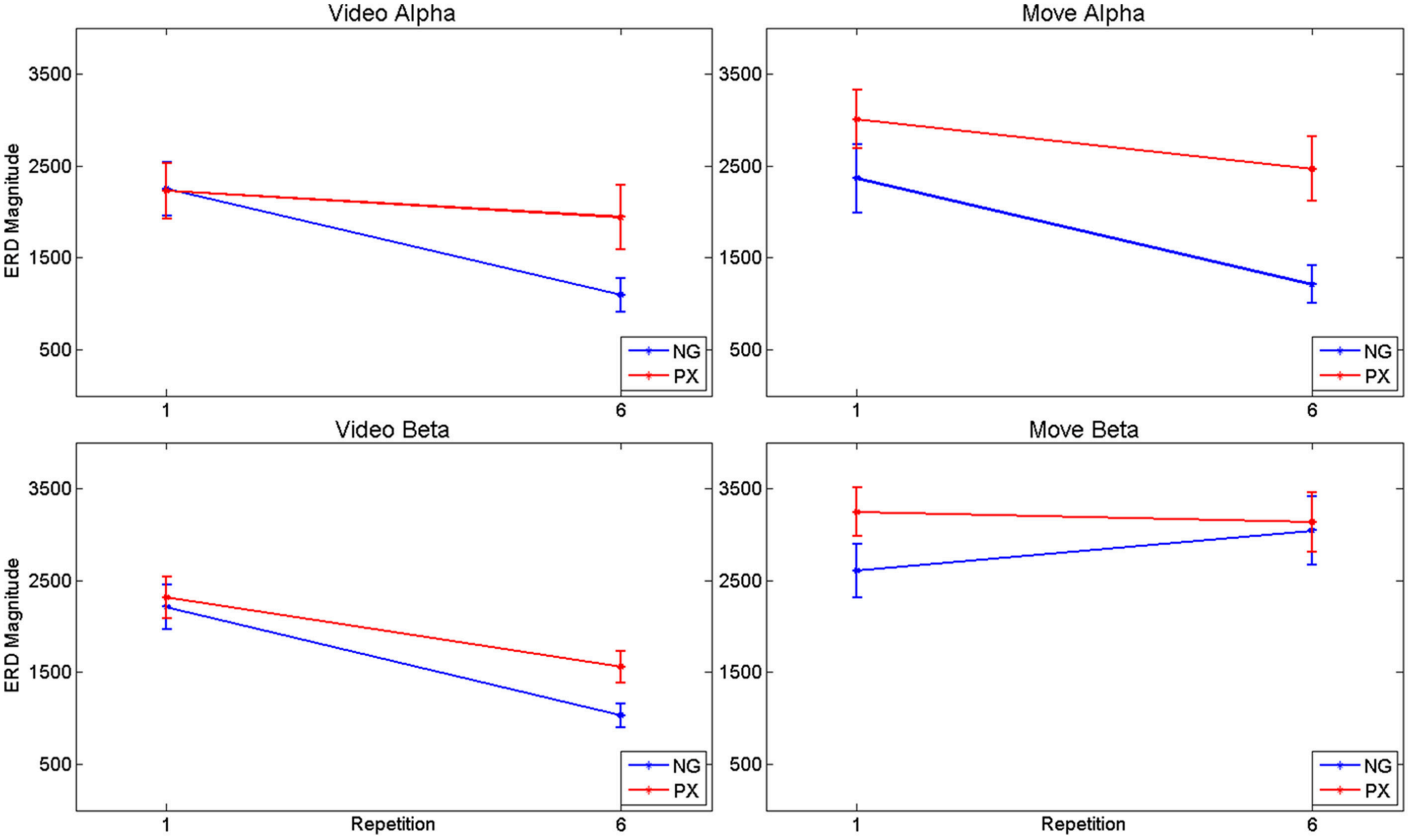
**ANOVA results for alpha and beta ERD, separately for video observation and motor execution portions of the tasks**. In general, both tasks showed repetition-related decreases in magnitude of ERD, with significant interaction effects: the magnitude of repetition-related ERD decrease in the NG task was greater than in the PX task.

Consistent with our predictions, *t*-tests demonstrated a significant decrease amount of alpha ERD in the NG task from repitition 1 to repetition 6 in both the video (51.2%, *p* = 1.02 × 10^−5^) and movement (48.8%, *p* = 0.0002) phases of the experiment (Figures 5A–D). In the control, PX task, there were no significant changes in magnitude of alpha ERD in the video observation phase; there was a 17.9% decrease in the movement phase (*p* = 0.029; Figures 5E–H). The repeated measures ANOVA demonstrated an interaction between task and repetition in the video phase, with NG showing a greater decrease in alpha ERD than PX over repetitions [*F*_(1, 11)_ = 8.0; *p* = 0.015; $\eta_{p}^{2}=0.40$] (Figure 7, top-left). A similar interaction effect was observed for the movement phase: NG showed a greater decrease in alpha ERD than PX over repetitions [*F*_(1, 11)_ = 5.3; *p* = 0.041; $\eta_{p}^{2}=0.35$] (Figure 7, top-right). These results are consistent with our predictions that alpha ERD would decrease over repetition, and that the magntidue of repetition-related alpha ERD decrease would be greater in NG than PX.

In the beta band, *t*-tests demonstrated no significant changes in either task from M1 to M6 (Figures 6C,D,G,H), though changes were seen from V1 to V6. In NG, a 53.3% decrease was seen (*p* = 4.7 × 10^−5^; Figures 6A,B), while a 32.7% decrease was seen in PX (*p* = 0.0005) (Figures 6E,F). Finally, repeated measures ANOVA showed an interaction between task and repetition in both the video observation [*F*_(1, 11)_ = 9.83; *p* = 0.009; $\eta_{p}^{2}$ = 0.444] (Figure 6, bottom-left) and movement phases [*F*_(1, 11)_ = 5.21; *p* = 0.041; $\eta_{p}^{2}$ = 0.30] (Figure 6, bottom-right), with NG showing a greater decrease in alpha ERD than PX over repetitions. These results demonstrate that the overall pattern of beta ERD results were similar to the pattern of alpha ERD results, though no repetition-related change was seen during the motor phase.

In sum, the alpha ERD findings were consistent with our predictions: decreasing amounts of alpha ERD were seen over the course of practice, with a greater change in alpha ERD in NG than in PX. Beta ERD findings were overall similar in direction to the alpha ERD findings, though no repetition-related change was seen during the motor phase.

## Discussion

Our primary goals were (1) to demonstrate convergent validity (empiric evidence that two theoretically-related measures are indeed related) between the imitation of novel, meaningless gestures and overlearned praxis gestures, and (2) to characterize repetition effects associated with the practice of novel gestures, and (3) to assess the degree to which repetition of novel gestures was associated with similar physiological effects to the repetition of overlearned, praxis gestures.

With regard to the first goal, we demonstrated similar patterns in the two conditions of activation over scal topography and over alpha and beta frequency ranges. This correspondence is consistent with the notion that similar mechanisms are being activated in the NG task and the PX task, to the extent of the EEG's measurement.

With regard to the second goal, we demonstrated repetition-related decreases in magnitudes of alpha and beta ERD in the NG task. This is a non-trivial finding. While prior studies of motor skill learning have demonstrated constriction of activation, the vast majority of previous tasks were performed with tasks that are driven principally by the primary motor cortex (Kelly and Garavan, 2005). The current NG task, by contrast, activates regions similar to praxis function and therefore represents a novel investigation of motor learning.

With respect to the third goal, we found qualitatively similar topographical constriction of ERD associated with the repetition of the overlearned praxis gestures, though to a lesser degree seen with the novel gestures. Because progressive topographical constriction was seen with already-overlearned praxis gestures, these results are consistent with the notion that the physiological effects seen are due at least in part to attentional effects that are seen both when “learning” novel gestures and with repeating overlearned gestures. Petersen et al.'s “scaffolding hypothesis” provides a plausible explanation for this finding (Petersen et al., 1998). Under this account, early (i.e., within-session) practice is associated with progressive minimization of activation in regions that support effortful movement execution. This account is also consistent with the work of Dovern et al., who demonstrated that lesions of the praxis network specifically affect *intentional* (but not automatic) motor sequence learning (Dovern et al., 2011). In the context of the current results, one could imagine that participants would have to attend less carefully both to the video (repetition suppression) and to their own performance as they repeat (practice) both the overlearned and novel gestures.

It may be argued that the ability to perform a task with a decreased need for attentional resources may be precisely what is talked about when improved performance is discussed. Indeed, “automaticity” is typically defined by a declining need for attentional resources (Cohen et al., 1992; Wu et al., 2015). It is plausible that the PX gestures, while generally overlearned, are requested in a new setting for the individual. As such, the changing physiological responses over the course of the experiment reflect increasing automaticity in this context.

We note however that the size of the ERD practice effect was greater in the NG task than the PX task. One possible explanation is that the repetition effect on attention is greater in the NG task than the PX task. Another possibility is that an additional process related to skill learning is present in the NG task, beyond the attentional effect also seen in the PX task. The effect we describe may have implications for skill learning clinical populations. However, rather than manifesting in acquired apraxia, which typically occurs after most functional skills have already been learned, alterations of skill learning mechanisms may have more relevance to developmental disorders. Indeed, we view the disordered acquisition of complex motor skills in ASD as analogous—and perhaps caused by the same pathogenic mechanisms—as alterations of skill social and communicative skill learning (Mostofsky and Ewen, 2011). Ongoing research is examing the relationhsip between praxis-like motor skill acquisition and social/communicative competence in ASD.

The current work provides evidence to a number of claims: that our new novel-gesture-learning paradigm activates similar regions to those activated by performance of praxis gestures, and that repetition of the novel gestures leads to a decrease in alpha ERD. Nevertheless, there are limitations to broader interpretations. The examiation of such limitations provides a roadmap for future research. The first limitation is that the participants performed most gestures correctly on the first attempt. In our experience developing this task in neurotypical adults, it was challenging to develop gestures which were routinely performed correctly by the sixth repetition, but incorrectly on the first. It is possible that the similarity in practice effects (despite a difference in magnitude) was due to the fact that the gestures were often performed correctly on the first attempt. Stated another way, it is possible that a qualitatively different physiological practice effect may be seen during a task in which participants perform the task incorrectly before performing it correctly. Indeed, there are multiple phases to motor skill learning (Kelly and Garavan, 2005), and learning to perform a skill correctly may take place at a phase different from learning to perform it efficiently.

The second is that the measurement of “correct” vs. “incorrect” is based on human judgment. While future work may take advantage of more technology-based metrics, these metrics still require judgements about which variables to consider to be critical to judgements about correct performance and which variables are not to be considered.

A third limitation is that we did not focus on specific cortical areas. While we optimized our spatial analysis through the use of CSD, we nevertheless used scalp signals, which cannot reliably be related to specific cortical areas. Future work may involve source localization to identify the role of specific cortical areas of interest. In particular, given the role of attention in these results, it will be of future interest to examine activation in attentional/cognitive control regions (Petersen et al., 1998).

In summary, our results demonstrate that similar topographical and frequency patterns of activity are seen in this new novel-gesture-learning task as compared with repetition of overlearned praxis gestures. Further, we have shown a decrease in amount of alpha ERD as subjects practiced the novel gestures. Repetition of overlearned praxis gestures also showed a reduction in alpha ERD, though to a lesser extent than in the case of the overlearned praxis gestures. We speculate that a reduction in alpha ERD is related to decreased need for attentional resources to be devoted to the task.

## Author contributions

JE, AP, BL, KA, MH, NC, and SM contributed to the design of the experiments. AP, DM, BL, and KA contributed to the acquisition of data. JE, AP, DM, and BL contributed to the analysis of data. JE, AP, DM, BL, MH, NC, and SM contributed to the interpretation of the data. All authors reviewed the manuscript for important intellectual content.

## Funding

This work was funded by the National Institute of Neurological Disorders and Stroke/National Institutes of Health (Intramural Program – MH; K23NS073626 and R21NS091569 to JE; and R01NS048527 to SM) and Autism Speaks (to SM).

### Conflict of interest statement

The authors declare that the research was conducted in the absence of any commercial or financial relationships that could be construed as a potential conflict of interest.
